# Fabrication and Evaluation of Electrospun Silk Fibroin/Halloysite Nanotube Biomaterials for Soft Tissue Regeneration

**DOI:** 10.3390/polym14153004

**Published:** 2022-07-25

**Authors:** Soheila Mohammadzadehmoghadam, Catherine F. LeGrand, Chee-Wai Wong, Beverley F. Kinnear, Yu Dong, Deirdre R. Coombe

**Affiliations:** 1 School of Civil and Mechanical Engineering, Curtin University, Bentley, WA 6102, Australia; sm.mohamadzade@gmail.com; 2 Curtin Health Innovation Research Institute, Faculty of Health Sciences, Curtin University, Bentley, WA 6102, Australia; catherine.legrand@postgrad.curtin.edu.au (C.F.L.); cwwon6@gmail.com (C.-W.W.); bfkinnear@gmail.com (B.F.K.); 3 Curtin Medical School, Pharmacy and Biomedical Sciences, Curtin University, Bentley, WA 6102, Australia

**Keywords:** silk fibroin, electrospinning, halloysite nanotubes, extracellular matrix, keratinocyte, myoblast, tissue engineering scaffolds

## Abstract

The production of nanofibrous materials for soft tissue repair that resemble extracellular matrices (ECMs) is challenging. Electrospinning uniquely produces scaffolds resembling the ultrastructure of natural ECMs. Herein, electrospinning was used to fabricate *Bombyx mori* silk fibroin (SF) and SF/halloysite nanotube (HNT) composite scaffolds. Different HNT loadings were examined, but 1 wt% HNTs enhanced scaffold hydrophilicity and water uptake capacity without loss of mechanical strength. The inclusion of 1 wt% HNTs in SF scaffolds also increased the scaffold’s thermal stability without altering the molecular structure of the SF, as revealed by thermogravimetric analyses and Fourier transform infrared spectroscopy (FTIR), respectively. SF/HNT 1 wt% composite scaffolds better supported the viability and spreading of 3T3 fibroblasts and the differentiation of C2C12 myoblasts into aligned myotubes. These scaffolds coated with decellularised ECM from 3T3 cells or primary human dermal fibroblasts (HDFs) supported the growth of primary human keratinocytes. However, SF/HNT 1 wt% composite scaffolds with HDF-derived ECM provided the best microenvironment, as on these, keratinocytes formed intact monolayers with an undifferentiated, basal cell phenotype. Our data indicate the merits of SF/HNT 1 wt% composite scaffolds for applications in soft tissue repair and the expansion of primary human keratinocytes for skin regeneration.

## 1. Introduction

Soft tissues are those that surround and support other tissues within the body. There are a variety of soft tissues, including muscle, tendon, adipose tissue, skin, blood vessels, and articular cartilage. They are well hydrated, and their cells exist within an extracellular matrix (ECM) composed primarily of fibrous collagens and elastin surrounded by glycoproteins and proteoglycans that facilitate hydration. Soft tissue damage can occur as a result of trauma, disease, aging, or surgery. Traditionally, soft tissue damage is treated by autologous tissue implantation. Still, this method has its challenges, in that implanted tissues may be reabsorbed, causing volume losses and scarring as well as donor site morbidity [1,2]. Hence, various biomaterials have been trialed for their use as scaffolds for soft tissue engineering. Ideally, the scaffolds should mimic naturally occurring ECM, a meshwork of fine fibres formed by collagens and elastin [2,3].

Electrospinning is a versatile technique for producing micro/nanofibre networks that resemble the ECM. The process entails a high electrical voltage applied to a polymer solution at a finite distance between a capillary and a collecting substrate. By careful selection of the material being electrospun and by adjusting parameters like voltage, needle-to-collector distance, and flow rate, it is possible to produce fibres ranging in diameter from low nanometres to hundreds of micrometres [3]. These nanofibrous scaffolds possess high surface area-to-volume ratios and high porosities reminiscent of the ECM, and it is these properties that facilitate cell attachment, as well as nutrient and waste exchange [4,5].

Scaffolds for soft tissue engineering should be biocompatible, biodegradable, trigger minimal inflammatory responses, and be able to be moulded into different shapes. Silk fibroin (SF), a protein obtained from the *Bombyx mori* silkworm, is used extensively to engineer scaffolds for repairing soft tissues owing to its high mechanical strength, cytocompatibility, and malleability [6,7]. Silk fibroin supports the adhesion and spreading of human keratinocytes, fibroblasts, and skeletal muscle myoblasts [6,8,9,10]. Our work with SF sponges [9] and other studies of electroactive SF scaffolds demonstrate the compatibility of SF for myoblast differentiation [6,11].

Extensive studies have been conducted using SF as a biomaterial for skin wound healing, and many are included in two recent reviews [7,12]. For example, Zhang et al. [13] used small and large animal models plus clinical evidence to demonstrate the efficacy of SF films for assisting the healing of full-thickness skin wounds. Others have explored using nanomatrices of electrospun SF as a dressing for burn wounds [14]. A number of SF-based materials for wound healing have been commercialised (e.g., products manufactured by Fibroheal Woundcare Pvt. Ltd. Bangalore Karnataka, India), but like the films used by Zhang et al. [13] and the nanomatrices used by Ju et al. [14], these are detachable dressings that facilitate healing rather than being scaffolds/implants. In contrast, Park et al. produced bilayered skin substitutes using electrospun SF nanofibrous scaffolds and air-liquid co-cultures of keratinocytes and fibroblasts [15], and Miguel et al. [16] prepared two layered SF-based electrospun membranes that resembled the epidermis and the dermis. In these studies, SF electrospinning processes were modified to increase pore sizes to allow better cell infiltration or to achieve porosities that resembled the targeted skin layer. In the latter study, this was achieved by making composites of SF and poly(caprolactone) for the epidermal layer and SF with hyaluronan for the dermis [16]. However, none of these studies addressed the significant clinical problem of limited primary human keratinocyte expansion in vitro due to terminal differentiation.

Despite numerous studies highlighting the benefits of SF as a favourable biomaterial for tissue regeneration, recent work has focused on SF composites, including SF/carbon nanotube composites, to achieve the desired functionality. For example, the presence of carbon nanotubes (CNTs) offers the option of tailoring the stiffness and strength of the SF composite according to the tissue application [17]. In addition, CNTs make SF composites conductive, meaning their use as a bioelectronic interface is possible in devices to control a neuron’s bioelectric activity [18]. However, the potential toxicity of CNTs is a great concern [19]. Accordingly, we examined whether the inclusion of halloysite nanotubes (HNTs) in electrospun scaffolds of SF would improve their functionality. HNTs are double-layered aluminosilicates that occur naturally as hollow tubular structures with aggregated particle sizes generally in a submicron range [20]. These nanotubes are a safe and biocompatible material, and their biomedical applications, particularly in the area of sustained drug release, have been highlighted in reviews [20,21,22]. HNTs can improve the mechanical and thermal properties, as well as the drug-loading properties of polymers [23,24]. Including HNTs in gelatine scaffolds prepared for bone regeneration improved the mechanical properties of elasticity and strength and their hydrophilicity [25]. This was also the case when electrospun scaffolds of polycaprolactone/gelatine contained various quantities of HNTs [26], and the importance of HNT inclusion for the extended release of the antibacterial metronidazole was demonstrated. Robust mechanical properties coupled with sustained antibacterial protection are consistent outcomes of HNT-reinforced electrospun nanofibrous scaffolds of materials like alginate [27], poly(L-lactide) [28], and poly(lactic-co-glycolic acid) [29] that were loaded with antibacterial drugs, and where tested, the electrospun HNT-containing scaffolds are non-cytotoxic and support cell growth [27,30]. Thus, including HNTs in SF electrospun scaffolds should facilitate the sustained release of antibacterial drugs, enhancing scaffold usefulness in vitro and in vivo. In addition, it is likely that hydrophilicity will be improved, meaning HNT-functionalised SF scaffolds may better support cell growth than SF-only scaffolds.

To our knowledge, no studies have reported the physical properties and cell compatibility of electrospun SF/HNT composite scaffolds. Herein, we describe the fabrication of electrospun SF/HNT composite scaffolds using different HNT loadings. The morphology, structure, hydrophilicity, thermal, and mechanical properties of these scaffolds are described. The scaffolds were assessed for their ability to support cell growth to determine if their use in repairing soft tissues, like skin and skeletal muscle, is feasible. Previously we demonstrated that SF sponges became coated with ECM proteins from adherent cells [9]. In the present study, scaffolds displaying the best cytocompatibility were chosen to examine the ECM deposited by fibroblasts onto these scaffolds. Our work, and that of others, has demonstrated the importance of the ECM for directing and regulating cell proliferation and differentiation [31,32,33], and scaffolds coated with decellularised ECM better regulate cell behaviour than their counterparts that lacked an ECM [33,34,35]. Here, we show that SF/HNT composite scaffolds coated with fibroblast ECM maintained primary human keratinocytes in a basal cell phenotype. This is potentially a clinically important finding. As these scaffolds markedly promoted the rapid expansion of keratinocyte populations in vitro, their use in hospital settings would benefit burn patients when the number of keratinocytes available for grafting can be critical for good patient outcomes. Collectively, our findings indicate that SF/HNT composite scaffolds are worthy of further investigation for soft tissue repair applications.

## 2. Materials and Methods

Preparation of SF. *Bombyx mori* SF (The Yarn Tree, Greenville, SC, USA) was extracted from cocoons, as described by Rockwood et al. [36]. After discarding the silkworms, 5 g of cocoons were cut and boiled in a 0.02 M sodium carbonate, aqueous solution (≥99%, Sigma Aldrich, North Ryde, Australia) for 30 min, and then rinsed with distilled water to remove the sericin proteins. The extracted SF was dried at room temperature (RT) for 24 h, then dissolved in 9.3 M lithium bromide solution (≥99.9%, Sigma Aldrich, North Ryde, Australia) at 60 °C for 4 h. This solution was dialysed against distilled water at RT for 48 h (dialysis membrane: MWCO 12400 Da, Sigma Aldrich) and filtered through a 22 µm membrane (Thermo Fisher Scientific, Waltham, MA, USA). This final solution was freeze-dried to produce regenerated SF.

Fabrication of Nanofibre Scaffolds. HNTs (donated by Imerys Tableware Asia Ltd., Kaeo, New Zealand) containing 49.5% SiO_2_, 35.5% Al_2_O_3_, 0.29% Fe_2_O_3_, 0.09% TiO_2_, and traces of CaO, MgO, K_2_O and Na_2_O were vacuum dried at 80 °C for 12 h. HNTs at different loadings (1, 3, 5, and 7 wt%) relative to the SF were dispersed in 98% formic acid (Sigma Aldrich) using an IKA T 25 ULTRA-TURRAX^®^ dispenser (IKA, Staufen, Germany) with a rotor speed of 7000 rpm for 30 min. This was followed by ultrasonication (ELMA Ti-H-5, Elma Schmidbauer GmbH, Singen, Germany) at 25 kHz with a sweep mode and 100% power intensity at 40 °C for 1 h. The lyophilised SF at a concentration of 13 wt%/v was added to the HNT/formic acid suspension and stirred for 3 h. Electrospinning was conducted at RT using a NaBond NEU nanofibre electrospinning unit (NaBond Technologies Co., Ltd., Shenzhen, China). Prepared SF/HNT solutions were loaded into a plastic syringe with an 18-gauge stainless steel needle. A voltage of 16 kV was applied to the blunt needle (inner diameter of 0.6 mm), and the flow rate was set at 0.3 mL/h by a syringe pump. The fibres formed on a plate collector covered with aluminum foil, and the needle-to-collector distance was 13 cm. Immersion in methanol (≥99.9%, Sigma Aldrich) for 15 min prior to air-drying increased fibre stability.

Scanning Electron Microscopy of Scaffolds. Nanofibre morphology was investigated using a Scanning Electron Microscope (SEM) (ZEISS EVO 40XVP, Carl Zeiss, Oberkochen, Germany). Samples were sputter coated with platinum and visualised at an accelerating voltage of 15 kV. Using ImageJ software (NIH, Bethesda, MD, USA), fibre diameters were determined by randomly measuring 100 fibres from each SEM image and calculating mean ± standard deviation (SD). To identify embedded HNTs in SF nanofibres, elemental analysis was performed on two replicates from each group of scaffolds using an Oxford Instruments (Abingdon, UK) energy-dispersive X-ray spectrometer (EDS) at an accelerating voltage of 10 kV. Analyses of EDS data were carried out using Inca software (software from Oxford Instruments).

Fourier Transform Infrared Spectroscopy. Chemical characterisation of the SF and SF/HNT composite scaffolds was carried out using Fourier Transform Infrared Spectroscopy (FTIR) (Spectrum 100 Optica FTIR Spectrometer, PerkinElmer Inc., Waltham, MA, USA). FTIR spectra data were acquired in a transmission mode with a 4 cm^−1^ resolution in a wave number range of 400–4000 cm^−1^ using attenuated total reflectance (ATR) from three replicate scaffolds for each group.

X-ray Diffraction Analysis. X-ray diffraction (XRD) was conducted on a D8 Advance X-ray diffractometer (Bruker AXS GmbH, Karlsruhe, Germany) with a Cu Kα radiation source (*λ* = 0.1541 nm) at 40 kV and 40 mA using a LynxEye detector (Bruker AXS). All samples were scanned with the diffraction angle 2*θ* = 5–40° at a scan rate of 0.015°/s. The d-spacing (*d*) for a specific scattering angle (*θ*) was determined according to Bragg’s equation, where *n* is an integer:

(1) nλ=2dsinθ

Contact Angle and Water Uptake Capacity. Scaffold hydrophilicity was evaluated by measuring the contact angles of a water droplet on the SF and SF/HNT composite scaffolds using a KSV CAM 101 Goniometer (KSV Instruments Ltd., Helsinki, Finland). A 5 µL water droplet was deposited onto the scaffold surface, and droplet images were captured automatically as a function of time. With the aid of CASTTM2.0 analysing software (USA KINO Industry Co. Ltd., Boston, MA, USA), the average contact angle was determined from 5 replicate images. The water uptake capacity (WUC) of SF/HNT composite scaffolds was determined by calculating the weight change between dry samples and samples that had been immersed in distilled water at RT for 24 h. Before measuring the weight of wet samples, excess surface water was removed using filter paper. The water uptake of the scaffolds was calculated as:

(2) $$\mathrm{water}\;\mathrm{uptake}\;\left(\%\right)=\left(\frac{W-W_{0}}{W_{0}}\right)\times 100$$

where *W* and *W*_0_ represent the weights of the samples before and after water immersion, respectively. Data are expressed as the average of 5 replicates.

BET Surface Area Analysis. The Brunauer−Emmet−Teller (BET) surface area of SF/HNT composite scaffolds was characterised using a Tristar 3000 (Micromeritics, Norcross, GA, USA), with the analysis being performed in the relative pressure (P/P0) range of 0.01–0.99.

Tensile Testing. Mechanical properties of SF/HNT composite scaffolds were assessed from stress-strain curves using a Lloyds Universal Testing Machine (Lloyds EZ50, Ametek-Lloyd Instruments Inc., Fareham, UK) at a crosshead speed of 10 mm/min and an ambient temperature of 25 °C with 65% humidity. Rectangular pieces of the scaffolds were attached to the testing machine as described by Chen et al. [37]. Rectangular cardboard frames (external 30 × 50 mm and internal 10 × 30 mm dimensions) were cut. Scaffolds were cut into 10 × 50 mm rectangles and the short edges attached to those of a frame, leaving an unsupported scaffold of dimensions 10 mm (width) × 30 mm (gauge length). A frame and attached scaffold were mounted on the testing machine, and the long edges of the cardboard frame were cut prior to each test. Scaffold thickness was measured using a micrometre at five positions, and a thickness range of 200–530 µm was recorded for tensile strength calculations.

Thermal Analysis. Thermogravimetric analysis (TGA) was performed using a Mettler-Toledo TGA/DSC 1 STARe System thermogravimetric analyser (Mettler-Toledo, Schwerzenbach, Switzerland), and the data were analysed with the aid of STARe software (Mettler-Toledo). Scaffold samples (8 mg) were placed into alumina crucibles and heated from 30 °C to 800 °C at a rate of 10 °C/min under the flow of argon gas at a rate of 20 mL/min. Measurements were performed on two replicate samples from each scaffold.

Cell Culture. The murine fibroblast cell line, 3T3 (European Collection of Cell Cultures, Porton Down, UK), was maintained in RPMI–1640 medium supplemented with 10% (*v*/*v*) fetal bovine serum (FBS, Serana Europe GmBH; Pessin, Germany), 10 mM HEPES, 1 mM sodium pyruvate and 2 mM glutamine (all from Gibco, Waltham, MA, USA; Life Technologies, Carlsbad, CA, USA) (RPMI/10% FBS). The murine myoblast cell line, C2C12 (American Type Culture Collection (ATCC), Manassas, VA, USA), was maintained in Dulbecco’s Modified Eagle Medium (DMEM; Gibco) containing 10% (*v/v*) FBS, 10 mM HEPES, 1 mM sodium pyruvate and 2 mM glutamine (DMEM/10% FBS). C2C12 cells were passaged at 60–70% confluency, with cells less than 20 passages being used. Primary human dermal fibroblasts (HDFs) (ATCC Cat# PCS-201-012) were maintained in DMEM/10% FBS and cells from passages 7–15 were used. Human neonatal keratinocytes (Gibco) were maintained in Defined Keratinocyte Serum Free Medium (DKSFM; Gibco) and cultured on tissue culture plastic coated with 3 μg/cm^2^ of collagen I (Sigma Aldrich) diluted in Phosphate Buffered Saline (PBS, Gibco). Keratinocytes at passage 4 or 5 were used in experiments. All cells were incubated in a humidified 37 °C incubator, equilibrated at 5% CO_2_. Cells were detached from their culture surfaces using 0.05% trypsin-EDTA (Gibco) and harvested into their designated media before being diluted to a specific concentration.

Antibodies. The rabbit polyclonal primary antibodies used were anti-collagen I, anti-collagen IV, and anti-fibronectin from Abcam (Cambridge, UK). The mouse monoclonal antibodies (mAbs) used were anti-myosin (clone NOQ7.5.4D, Millipore, Temecula, CA, USA), anti-involucrin (clone SY5; Sigma Aldrich), anti-keratin 10 (K10; clone LH2) and anti-keratin 14 (K14; clone LL001). Clones LH2 and LL001 were produced in the labs of E.B. Lane and I.M. Leigh [38,39] and were provided by Prof. Birgit Lane of A*STAR Institute of Medical Biology, Singapore. Secondary antibodies were Alexa Fluor 647 anti-rabbit IgG (Invitrogen, Thermo Fisher Scientific, Waltham, MA, USA), Alexa Fluor 488 anti-mouse IgG, Alexa Fluor 546 anti-mouse IgG, and Alexa Fluor 488 anti-rabbit IgG (Molecular Probes, Thermo Fisher Scientific, Waltham, MA, USA).

Proliferation Assay. A CellTiter-Blue assay (Promega, Madison, WI, USA) was used. Murine 3T3 and C2C12 cells were seeded onto ultra-violet (UV) sterilised 6 mm discs of SF and SF/HNT composite scaffolds. Scaffold discs were equilibrated in DMEM/10% FBS for 1 h before seeding with a 5 μL cell suspension containing 40 × 10^4^ (3T3) or 22 × 10^4^ (C2C12) cells/mL. Cells adhered for 30 min at 37 °C, then 2 mL of culture medium was added, and culturing continued for 1 or 3 days. Afterwards, the scaffold discs were transferred into wells of a 96-well black plate where 100 μL/well of phenol red-free DMEM medium and 20 μL/well of CellTiter-Blue reagent were added. The plate was incubated at 37 °C for 4 h, and fluorescence intensity was measured at wavelengths of 560 nm (excitation) and 590 nm (emission) using an EnSpire Multimode Plate Reader (Perkin Elmer, Waltham, MA, USA). A standard curve was generated using a known number of cells (20 × 10^4^ to 3 × 10^3^ cells/well) in 100 μL of phenol red-free DMEM added to a 96-well black plate, labelled with 20 μL/well of CellTiter-Blue reagent, incubated, and measured as described.

Immunocytochemistry. Cells cultured on 6 mm SF/HNT composite scaffold discs in 24-well tissue culture plates were fixed with 4% paraformaldehyde/PBS for 15 min, permeabilised with 0.1% Triton X-100 (Sigma Aldrich) in PBS for 3 min, and incubated in blocking buffer (PBS/10% (*v/v*) goat serum (Gibco)/1% (*v/v*) bovine serum albumin (BSA; Hyclone, Logan, UT)) for 1 h at RT. Primary antibodies diluted in blocking buffer were added and incubated for 1 h at RT. Cells were washed with PBS (3 × 5 min), then incubated for 1 h with an appropriate secondary antibody diluted in blocking buffer. Cells were washed with PBS, then incubated with 4,6-diamidino-2-phenylindole (DAPI; BD Pharmingen, Franklin Lakes, NJ, USA) diluted 1:2000 in PBS for 10 min. Following a PBS wash, scaffold discs were mounted on glass slides using Vectashield Mounting Medium (Vector Laboratories; Peterborough, UK). Immunofluorescent images were acquired on a Nikon A1+ Confocal Microscope (Nikon, Tokyo, Japan), and NIS-Elements version 4.20 software (Nikon Instruments Inc., Melville, NY, USA) was used. Images were processed using Fiji software [40]. Cells were also visualised by staining for 1 h at RT with Alexa Fluor 488 conjugated phalloidin (Molecular Probes, Thermo Fisher Scientific, Waltham, MA, USA) diluted in a blocking buffer. Cell nuclei were stained with DAPI, scaffolds were mounted, and cells were imaged as described.

Scanning Electron Microscopy of 3T3 Cells on Scaffolds. Cell morphology was assessed after days 1 and 3 of culture using a field emission scanning electron microscope (FE-SEM, Zeiss NEON 40 EsB Cross Beam). In a 6-well tissue culture plate, 3T3 cells were seeded onto 1 cm^2^ SF/HNT composite scaffolds (4 × 10^3^ cells/scaffold). After culturing, the samples were dehydrated by serial dilution in ethanol (10%, 30%, 70%, 90%, 100% (*v/v*) in distilled water) for 10 min at each concentration, then freeze-dried and coated with platinum. The samples were observed using FE–SEM at an accelerating voltage of 5 kV.

C2C12 Cell Differentiation. Scaffolds (1 cm^2^ SF/HNT) pre-incubated in DMEM were seeded with C2C12 cells (4 × 10^3^ cells/scaffold) and cultured for 4 days in phenol red-free DMEM/10% FBS. Differentiation medium of phenol red-free DMEM containing 10 mM HEPES, 1 mM sodium pyruvate, 2 mM glutamine, and 2% (*v/v*) horse serum (HS) (Invitrogen, Thermo Fisher) was applied, and the cells were cultured for 4 days, with the medium being replaced on day 2 of differentiation. Scaffolds were processed for immunocytochemistry and stained (1 h at RT) with the mouse primary mAb NOQ7.5.4D to detect muscle myosin. Scaffolds were then washed and incubated with Alexa Fluor 488 anti-mouse IgG secondary antibody for 1 h. After washing, the samples were incubated with DAPI, mounted, and imaged using a Nikon A1+ confocal microscope.

Fibroblast Derived ECM. UV sterilized, 6 mm discs of SF and SF/HNT 1 wt% composite scaffolds were placed in wells of a 24-well tissue culture plate, then seeded with a 5 µL cell suspension of 3T3 cells (50 × 10^4^ cells/mL) or HDFs (200 × 10^4^ cells/mL) and incubated at 37 °C for 30 min for cell adhesion. Then 1 mL DMEM (phenol red free)/10% FBS supplemented with 30 μg/mL ascorbic acid (Wako Pure Chemical Industries Ltd., Osaka, Japan) was added, and the cells were cultured for 7 days with the medium changed every 48 h. After which the scaffolds were rinsed with PBS and decellularised using Phospholipase A_2_ (PLA_2_, Sigma Aldrich) as reported [32]. Samples were incubated for 30 min at 37 °C with PLA_2_ (20 U/mL) in 50 mM Tris-HCl (pH 8), 0.15 M NaCl, 1 mM MgCl_2_, 1 mM CaCl_2_ (reagents from Sigma Aldrich), 0.5% sodium deoxycholate and 1× EDTA-free protease inhibitor (Roche, Basel, Switzerland). Treated samples were washed with PBS and then incubated for 30 min at 37 °C with 0.02 mg/mL DNase I (VWR Life Science Amresco, Solon, OH, USA) in 10 mM Tris-HCl (pH 7), 2.5 mM MgCl_2_, and 0.5 mM CaCl_2_ and finally washed in PBS.

Immunofluorescent Staining of ECM. ECM-coated scaffolds were processed for immunocytochemistry and incubated (1 h at RT) with antibodies (diluted in blocking buffer) recognising either fibronectin, collagen I, or collagen IV. After PBS washes, scaffolds were similarly incubated with an Alexa Fluor 647 anti-rabbit antibody diluted (1:400) in blocking buffer. Scaffolds were washed, mounted on glass slides, and imaged using a Nikon A1+ confocal microscope.

Keratinocyte Growth on Scaffolds. Keratinocytes in DKSFM were seeded (4 × 10^3^ cells/scaffold) onto 6 mm scaffold discs of SF and SF/HNT 1 wt% composites coated with 3T3 or HDF-derived ECM. The cells adhered for 30 min at 37 °C, then 1 mL DKSFM was added and incubated in a humidified 37 °C incubator, with medium changes every 48 h. On days 4 and 8 of culture, keratinocytes were processed and immunostained.

Graphing and Statistical Analysis. Quantitative data analyses were performed using GraphPad Prism 6 software (GraphPad, San Diego, CA, USA) and presented as a mean ± SD. Statistical comparisons of normally distributed data were a one-way analysis of variance (ANOVA) followed by Tukey’s post hoc test. Other data were analysed using a Kruskal-Wallis one-way ANOVA and a Dunn’s multiple comparison test. In all cases, *p* < 0.05 was considered statistically significant.

## 3. Results

### 3.1. Fibre Morphology

**Figure 1:**
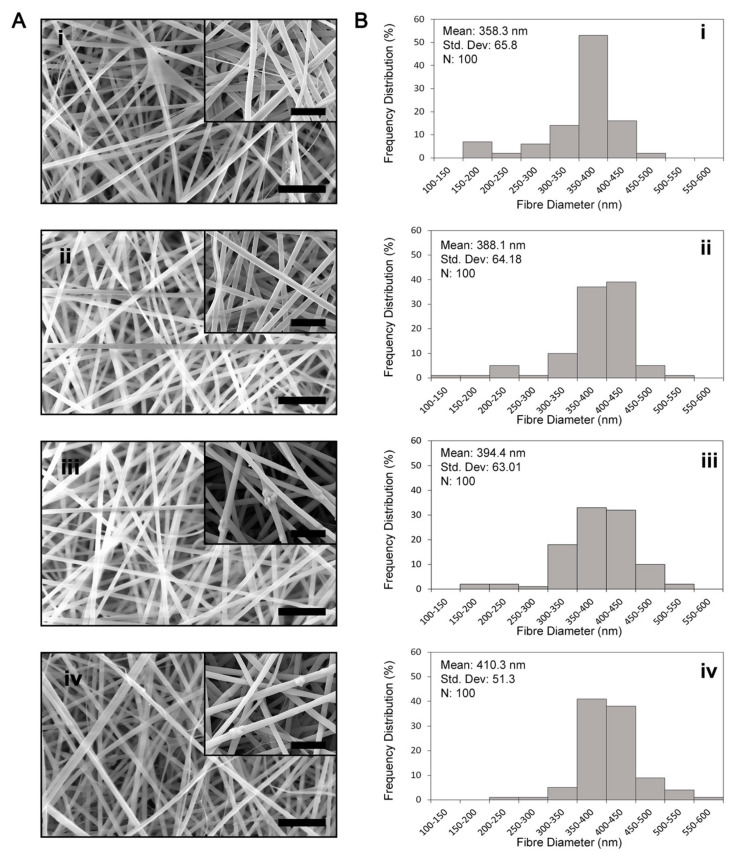

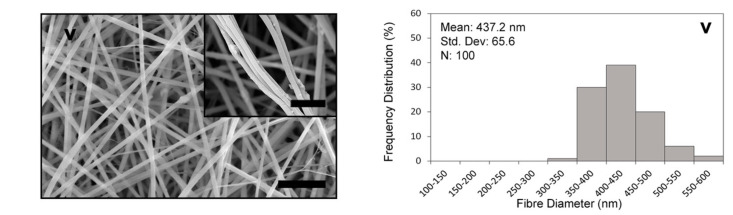
Morphology and diameters of electrospun fibres. (**A**) SEM images of SF/HNT composite scaffolds: (**i**) SF, (**ii**) SF/HNT 1 wt%, (**iii**) SF/HNT 3 wt%, (**iv**) SF/HNT 5 wt%, (**v**) SF/HNT 7 wt%; scale bar: 4 μm. Insets: high magnification images; scale bar: 1 μm. (**B**) Fibre diameter quantification: the percent of fibres with diameters of various sizes is shown: (**i**) SF, (**ii**) SF/HNT 1 wt%, (**iii**) SF/HNT 3 wt%, (**iv**) SF/HNT 5 wt%, and (**v**) SF/HNT 7 wt%. By ANOVA and Tukey’s post hoc test, SF fibre diameters were significantly less than those of the composite scaffolds. Fibre diameters of SF/HNT 7 wt% scaffolds were significantly higher than those of the other scaffolds (*p* < 0.05).

The SEM images revealed that electrospun SF fibres have smooth, uniform surfaces (Figure 1A-i), and even at high magnification, irregularities, or fractures in the fibre surfaces, were not evident (Supplementary Figure S1C). In contrast, scaffolds containing 1 wt% and 3 wt% HNTs had intermittent irregularities at high magnification, while scaffolds containing 5 wt% and 7 wt% HNTs had more frequent irregularities. Often these irregularities appeared as a swelling or a lump in the SF fibre (Supplementary Figure S1A-i,A-ii), while in other places, it appeared that aggregated nanotubes were protruding from the side of SF fibres (Figure 1A-iv,A-v and Figure S1B-i). With increasing HNT content, there was a noticeable increase in the number of fibres with very rough, irregular, fractured surfaces. This was particularly apparent when the HNT content was 7 wt% (Supplementary Figure S1B). Including HNTs at any concentration increased fibre diameters (Figure 1B-i–B-v).

**Figure 2:**
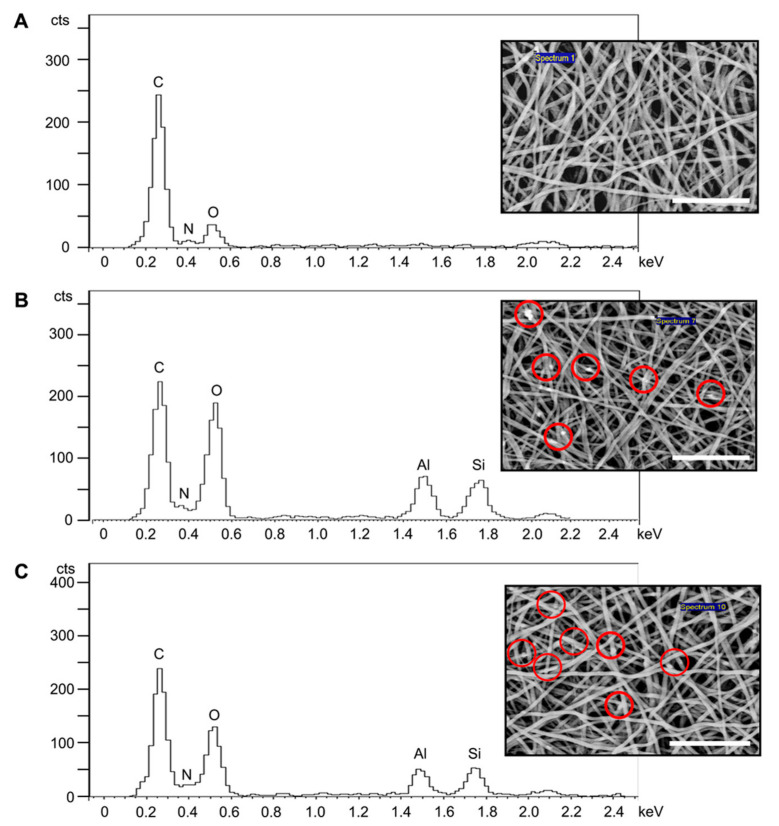

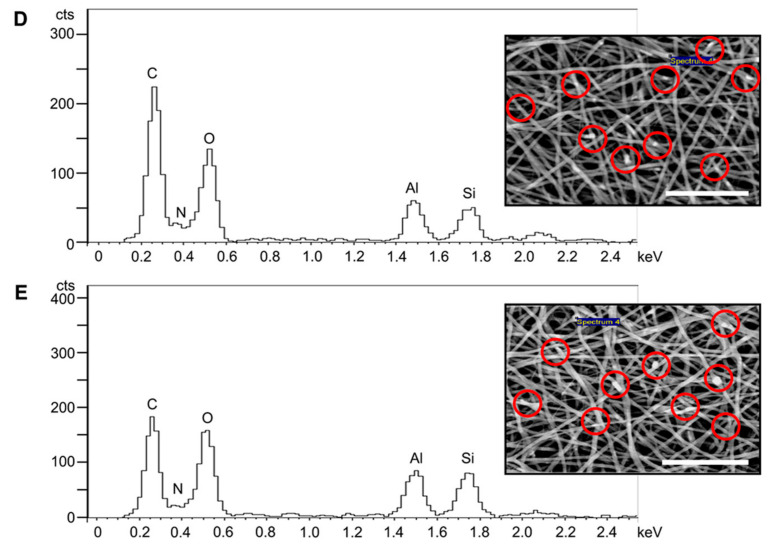
Identification of HNTs within the scaffolds. EDS spectra of SF/HNT composite scaffolds (**A**) SF, (**B**) SF/HNT 1 wt%, (**C**) SF/HNT 3 wt%, (**D**) SF/HNT 5 wt%, and (**E**) SF/HNT 7 wt%. Inserts: Scanning electron micrographs of the samples analysed. Red circles: HNTs incorporated into the SF fibres; scale bar: 10 μm.

The EDS spectra indicated that SF has a chemical composition of carbon, oxygen, and nitrogen (Figure 2A). The general chemical formula for halloysite clay is (Al_2_Si_2_O_5_(OH)_4_.nH_2_O), and the presence of aluminium and silicon elemental peaks in the SF solution prior to electrospinning was detected upon the addition of HNTs (Figure 2B–E). The SEM images (Figure 2 inserts) revealed that HNTs became incorporated into the SF fibres after electrospinning the SF/HNT solutions previously shown to have aluminium and silicon elemental peaks.

### 3.2. BET Surface Area

The measurements of BET surface areas of SF and SF/HNT composite scaffolds are summarised in Table 1. The BET-specific surface area of SF fibres was 4.47 m^2^/g, which increased as the HNT content increased, becoming 9.06 m^2^/g with the inclusion of 7 wt% HNTs.

### 3.3. Contact Angle and Water Uptake Capacity (WUC)

The contact angles measured on all scaffolds were less than 90° (Table 2). Scaffolds containing 1 wt% HNTs had a contact angle of 57.52° which is less than that of 64.23° for SF scaffolds. An increase in the HNT content beyond 1 wt% caused the contact angle to increase to that of SF scaffolds without HNTs, and scaffolds containing 7 wt% HNTs had a contact angle of 70.76°, which was significantly greater than the contact angle measured for SF scaffolds.

**Figure 3:**
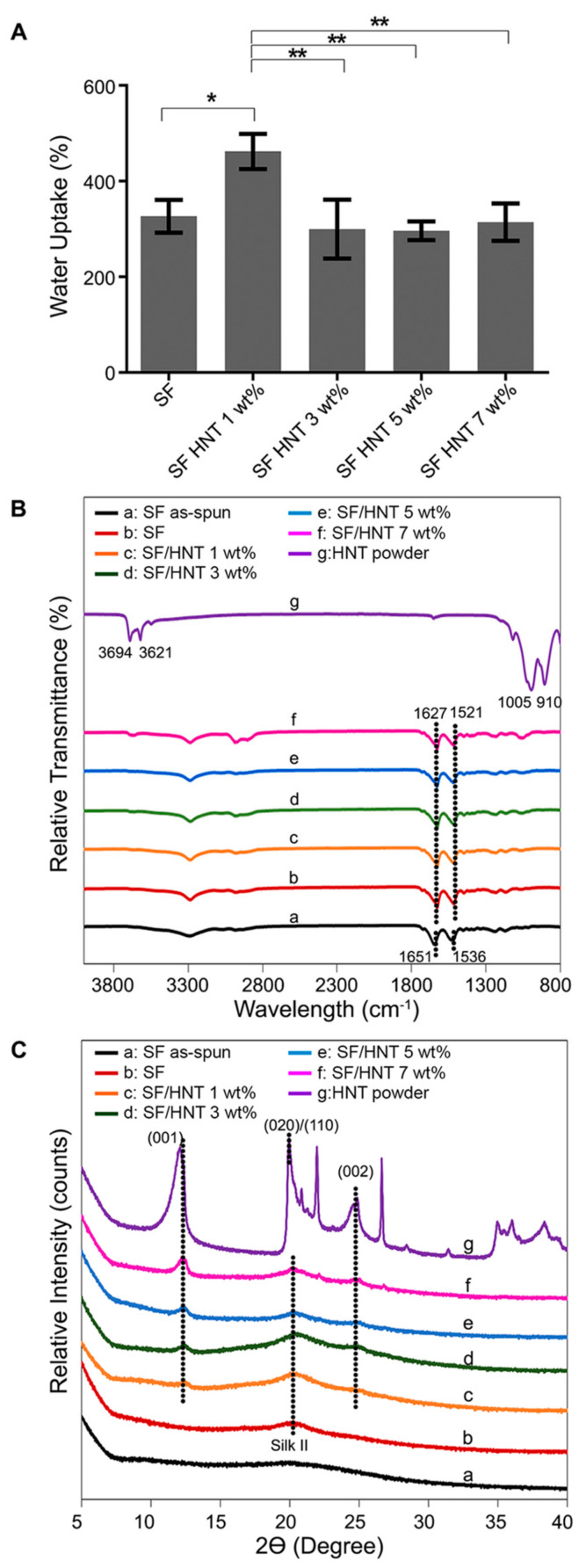
Characteristics of the scaffolds. (**A**) Water uptake capacity of SF scaffolds and SF/HNT composite scaffolds. All tests were performed on 5 replicates. Means ± SD are shown. Statistical analyses were ANOVA followed by Tukey’s test. * *p* ≤ 0.05, ** *p* ≤ 0.01. (**B**) FTIR spectra of as-spun SF scaffolds, methanol-treated SF scaffolds, and SF/HNT composite scaffolds. (**C**) XRD pattern of SF/HNT composite scaffolds and as-received HNTs.

Figure 3A shows the WUC data for all scaffolds after 24 h. For SF/HNT 1 wt% composite scaffolds, the measured WUC was 462% compared to 326% for SF scaffolds. Scaffolds containing 3 to 7 wt% HNTs had a measured WUC of between 290–314%. Overall, the WUC of SF/HNT 1 wt% composite scaffolds was significantly greater than that of the SF scaffolds and the other HNT-containing scaffolds.

### 3.4. FTIR Analysis

To determine whether the inclusion of HNTs into SF fibres altered SF molecular structures, FTIR analyses were performed (Figure 3B). In “as-spun” SF fibres the main peaks were detected at 1651 cm^−1^ (amide I) and 1536 cm^−1^ (amide II) [41]. These peaks can be attributed to the random coil conformation. After methanol treatment, the characteristic peaks of SF were found at 1627 and 1520 cm^−1^; these peaks can be assigned to amide I and amide II in the β-sheet conformations [42]. Analysis of the HNT powder revealed prominent peaks at 910 and 1005 cm^−1^, and other peaks at 3621 and 3694 cm^−1^ [43]. The different HNT composite scaffolds produced the same absorption peaks as the SF scaffolds, indicating the SF molecular structure was not altered by the inclusion of HNTs.

### 3.5. XRD Analysis

The XRD analysis (Figure 3C) for HNT powders shows major diffraction peaks at 2θ angles of 12.49°, 20.44°, and 25.04°, which were assigned to (001), (020)/(110), and (002) crystal planes, respectively [44]. Based on Bragg’s law, these peaks are associated with *d*-spacing values of approximately 0.73, 0.44, and 0.35 nm, respectively. The amorphous structure of “as-spun” SF did not have an XRD peak. However, the SF peaked at 2θ = 20.3° after methanol treatment. This peak was observed for all scaffolds regardless of their HNT content. The SF peak at 2θ = 20.3° overlaps with the diffraction pattern of HNTs at 20.44°. This observation means it cannot be solely attributed to either SF or HNTs. In all composite scaffolds, the HNT peak pattern was apparent, and as the HNT’s content increased, the diffraction patterns at 2θ angles of 12.5° and 25.1° intensified. The *d*-spacing values for these two peaks remained unchanged at 0.7 and 0.35 nm regardless of the HNT content.

### 3.6. Mechanical Properties

**Figure 4:**
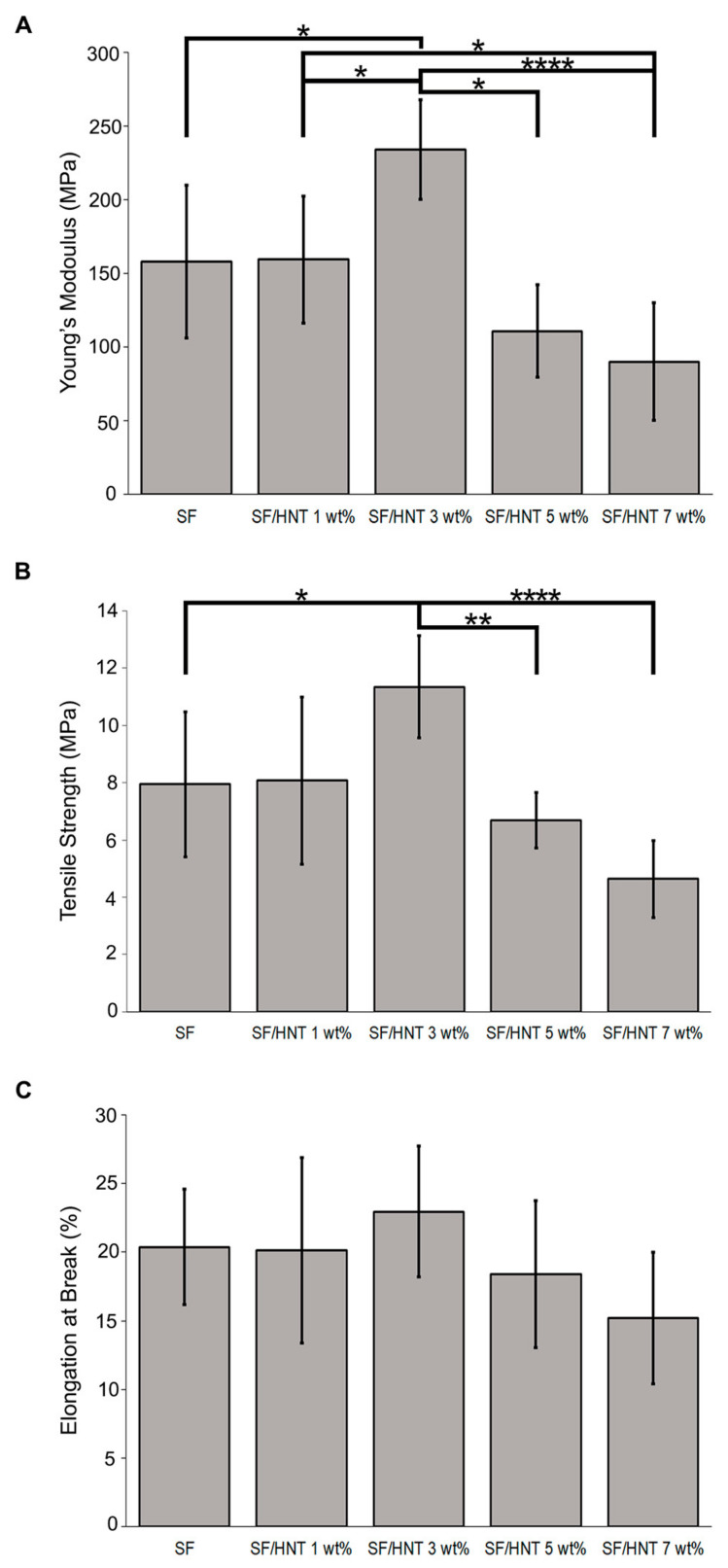
Mechanical properties of SF and SF/HNT composite scaffolds. (**A**) Young’s modulus, (**B**) tensile strength, and (**C**) elongation at break of SF. The analyses were performed on 10 replicates for each batch of scaffolds. Means ± SD are shown. Statistical analyses were ANOVA followed by Tukey’s test. * *p* ≤ 0.05, ** *p* ≤ 0.01, **** *p* ≤ 0.0001. Elongation at break data was not significantly different.

Neat SF scaffolds had a Young’s modulus of 158.08 ± 51.82 MPa and a tensile strength of 7.94 ± 2.54 MPa (Figure 4A,B). While the addition of 1 wt% HNTs did not significantly alter these values, scaffolds containing 3 wt% HNTs had a significant increase in Young’s modulus at 233.67 ± 18.40 MPa and in tensile strength at 11.35 ± 1.79 MPa. Increasing the HNT content to 7 wt% reduced the Young’s modulus and tensile strength to 90.20 ± 40.07 MPa and 4.64 ± 1.34 MPa, respectively. A similar trend was observed in the elongation at break (Figure 4C). The value for SF scaffolds was 20.39%, while the maximum value of 22.95% occurred with scaffolds containing 3 wt% HNTs, but it decreased to 15.21% with 7 wt% HNTs.

### 3.7. Thermal Stability

**Figure 5:**
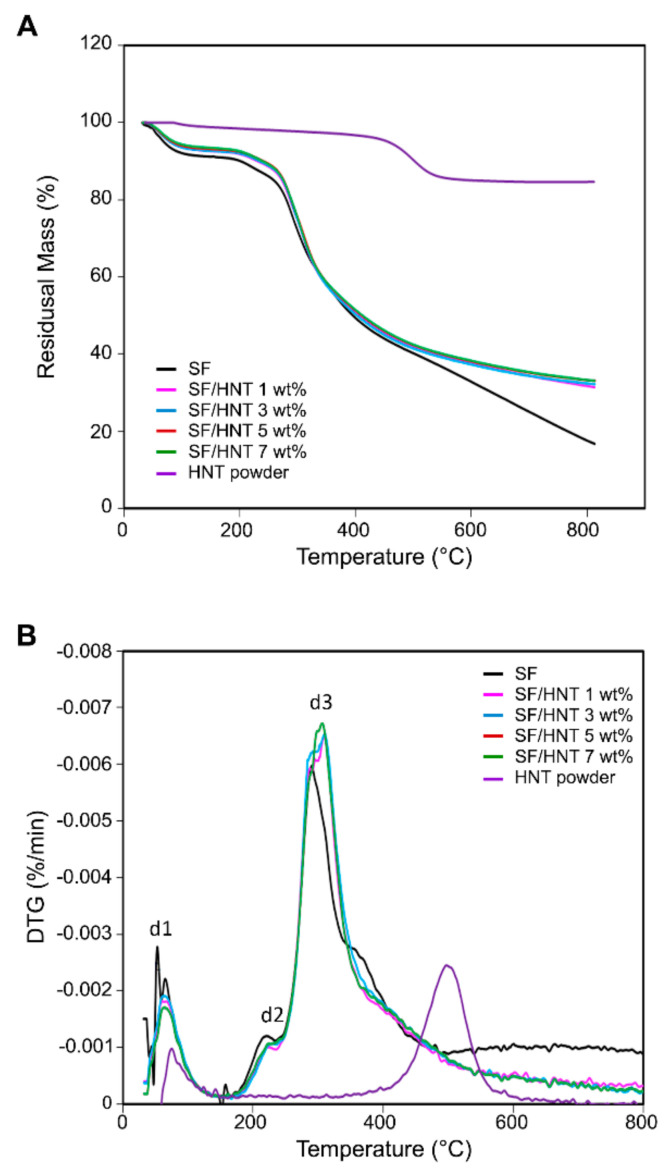
Comparison of thermal stability of SF scaffolds, SF/HNT composite scaffolds, and as-received HNTs: (**A**) TGA curves and (**B**) DTG, the three temperature peaks: d1, d2, and d3 are indicated. In both (**A**) and (**B**), the curves for SF/HNT 5 wt% are directly overlaid by SF/HNT 7 wt%, meaning only the latter is visible.

The effect of HNTs on the thermal decomposition/stability of SF/HNT composite scaffolds was investigated by TGA (Figure 5A and Table 3). For all scaffolds, thermogravimetric curves displayed two distinct phases, one below 100 °C and another at about 270–350 °C (Figure 5A). The first phase of weight loss can be attributed to water evaporation, while the second phase at 270–350 °C may be associated with fibroin degradation and the breakdown of peptide bonds [45]. The incorporation of HNTs increased the thermal stability of SF fibres, as the onset temperatures T_10%_ for SF/HNT composite scaffolds at 230–240 °C were greater than that of SF scaffolds at 201.65 °C. Similarly, as the HNT content increased, the T_50%_ increased from 395.3 °C for SF scaffolds to 407–412 °C for the composites. The highest temperatures recorded were for composite scaffolds containing 3 wt% HNTs, while scaffolds containing the higher HNT content of 5 and 7 wt% had a minor decrease in both T_10%_ and T_50%_ values (Table 3).

Derivative thermogravimetric (DTG) curves (Figure 5B) reveal three distinct temperature peaks, T_d1_, T_d2_, and T_d3_. For SF scaffolds, the T_d1_ peak at 68.22 °C is probably due to moisture loss, while the T_d3_ peak detected at 291.32 °C is more likely due to SF decomposition (Table 3). With the addition of HNTs, both T_d2_ and T_d3_ increased, reaching their maximum value for composite scaffolds containing 3 wt% HNTs, while for T_d1_, the maximum value was achieved for SF/HNT 1 wt% scaffolds. Similar to the TGA values, all three decomposition temperature peaks decreased as the HNT content in the composite scaffolds increased to 7 wt%, although their T_d3_ was still greater than that of SF.

### 3.8. Fibroblast Growth on SF and SF/HNT Composite Scaffolds

**Figure 6:**
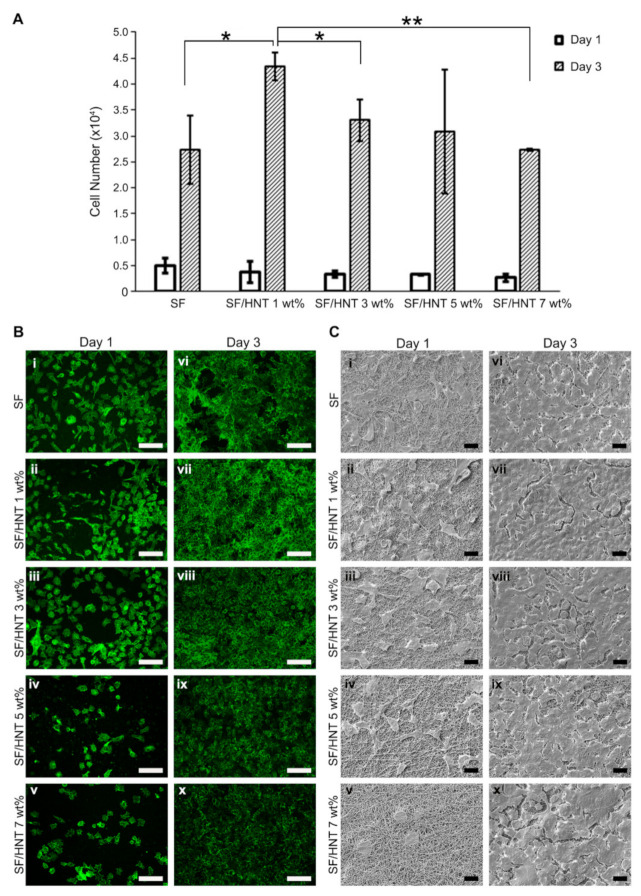
Fibroblasts proliferate on SF and SF/HNT composite scaffolds. (**A**) The numbers of 3T3 cells after 1 and 3 days of culture. Data are means ± SD of 6 replicates and represent three independent experiments. Statistical analyses were ANOVA with Fisher’s least significant difference test. * *p* ≤ 0.05; ** *p* ≤ 0.01. (**B**) Representative images of 3T3 cells cultured for 1 and 3 days on: SF (**i**,**vi**), SF/HNT 1 wt% (**ii**,**vii**), SF/HNT 3 wt% (**iii**,**viii**), SF/HNT 5 wt% (**iv**,**ix**) and SF/HNT 7 wt% (**v**,**x**), after staining with phalloidin-Alexa Fluor 488 (Green). Scale bar: 50 µm. (**C**) FE-SEM images of cells cultured for 1 and 3 days on: SF (**i**,**vi**), SF/HNT 1 wt% (**ii**,**vii**), SF/HNT 3 wt% (**iii**,**viii**), SF/HNT 5 wt% (**iv**,**ix**) and SF/HNT 7 wt% (**v**,**x**); scale bar: 10 µm.

To determine whether HNTs in SF scaffolds affected cell behaviour, the growth of 3T3 fibroblasts on the scaffolds was assessed using a CellTiter Blue assay. On all scaffolds, cell number increased with increasing time in culture. Cell numbers on the scaffolds on day 1 were similar, but on day 3, significantly more cells were recorded on SF/HNT 1 wt% composite scaffolds than on SF scaffolds (Figure 6A). Visualisation of the cells on the scaffolds after staining with Alexa Fluor 488 conjugated phalloidin indicated SF/HNT 1 wt% composite scaffolds best supported 3T3 cell growth at day 3 (Figure 6B). Although slightly fewer cells were visible on composite scaffolds containing 5 and 7 wt% HNTs, these cells were evenly distributed over the scaffolds, suggesting uniformity in the surface characteristics across the scaffolds. Day 1 images revealed the cell shape differed on the various scaffolds. Many cells on SF scaffolds and SF/HNT 1 wt% scaffolds were elongated and had long projections, indicating cell spreading (Figure 6B-i–B-ii). The cell shape changed as the HNT content increased, cells lost their elongated shape, and cells on scaffolds with the highest HNT content were almost spherical (Figure 6B-iii–B-v).

Representative FE-SEM micrographs of 3T3 cells on SF and SF/HNT composite scaffolds after 1 and 3 days of culture confirmed that all scaffolds provided an environment that supported cell attachment (Figure 6C). The cell shape on all scaffolds except SF/HNT 7 wt% scaffolds indicated adhesion and spreading had occurred by day 1. The cells on SF/HNT 7 wt% composites were round and not spread (Figure 6C-v). After 3 days of culture, the cell layers virtually covered the scaffold surface, except for small areas between the expanding cell colonies, but on SF/HNT 1 wt% composite scaffolds, the areas not occupied by cells appeared smaller than those on the other scaffolds (Figure 6C-vii). These data are consistent with the cell proliferation results (Figure 6A) and the confocal microscopy images (Figure 6B).

### 3.9. C2C12 Myoblasts Differentiate Differently on the Scaffolds

**Figure 7:**
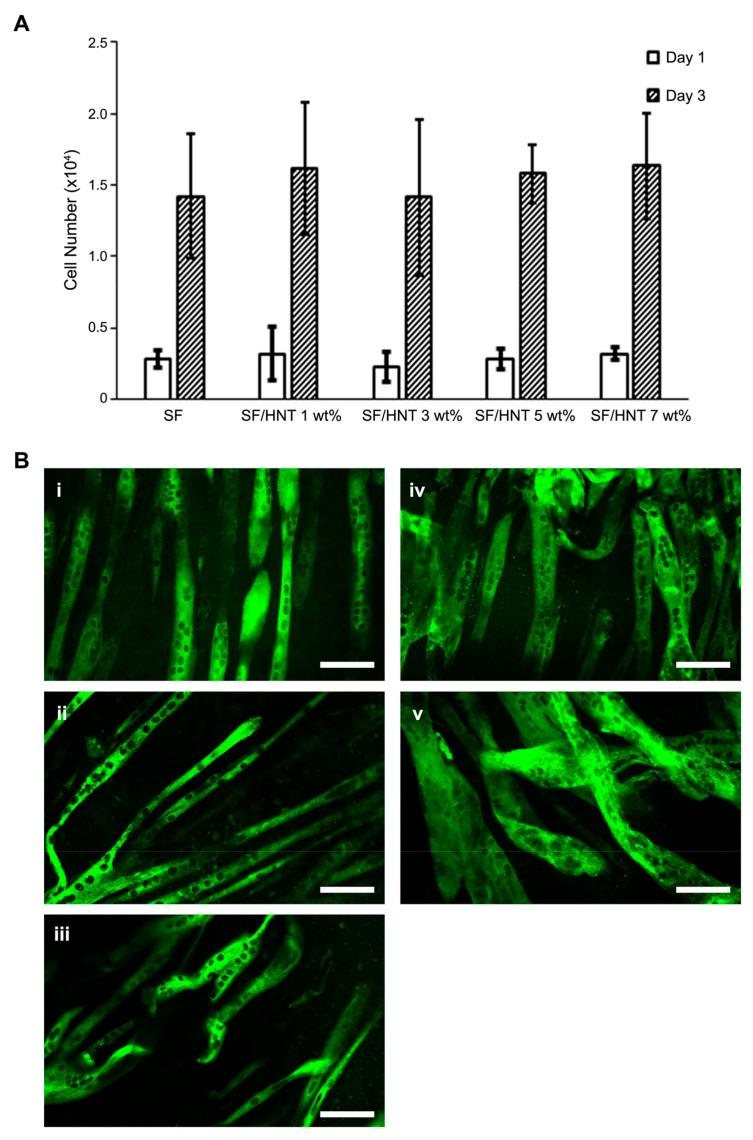
Myoblasts proliferate and differentiate on the scaffolds. (**A**) C2C12 myoblast proliferation on SF and SF/HNTs scaffolds after 1 and 3 days of culture. Data are means ± SD of 6 replicates. Data are representative of three independent experiments. Statistical analyses were ANOVA followed by Tukey’s test; *p* > 0.5, no significant differences were detected. (**B**) C2C12 cell differentiation. Cells were stained with an anti-myosin antibody (green) to reveal myotubes: SF (**i**), SF/HNT 1 wt% (**ii**), SF/HNT 3 wt% (**iii**), SF/HNT 5 wt% (**iv**), SF/HNT 7 wt% (**v**); scale bar: 100 μm.

We previously found that SF from different silkworm species influenced myoblast differentiation and the appearance of the resulting myotubes differently [9]. Thus, we asked whether myoblast differentiation would proceed equally well on scaffolds of *B. mori* SF that were functionalised with varying amounts of HNTs. Myoblast proliferation after three days of culture on the scaffolds was comparable regardless of scaffold HNT content (Figure 7A). To determine if C2C12 cell differentiation varied on the different scaffolds, cells were seeded on the scaffolds and allowed to proliferate to almost confluence and then were switched into a differentiation medium. Differentiated cells and myotube formation were visualised by staining with an anti-muscle myosin antibody. Myotube formation occurred on all scaffolds (Figure 7B-i–B-v), but the characteristics of the myotubes varied according to the scaffolds on which they were growing. Myotubes were longer, thinner, and better aligned on SF/HNT 1 wt% composite scaffolds than on any other composite scaffold, and their nuclei were organised in a single line (Figure 7B-ii). Myotubes that formed on scaffolds containing 3, 5, and 7 wt% HNTs were short, thick, and disorganised. This was very clear for myotubes on scaffolds containing 5 and 7 wt% HNTs (Figure 7B-iii–B-v). On SF scaffolds, myotubes were aligned, but they had disorganised nuclei and were shorter and thicker than those on SF/HNT 1 wt% composite scaffolds (Figure 7B-i,B-ii). Thus, SF/HNT 1 wt% composite scaffolds best supported C2C12 myotube formation.

### 3.10. Fibroblasts Deposit ECM on the Scaffolds

Previously we showed that primary human keratinocytes could be maintained in an undifferentiated state if cultured on fibroblast-derived ECM [32]. Here we asked whether an SF scaffold of electrospun fibres resembling an ECM could substitute for the natural decellularised matrix, or if an electrospun, SF substrate, functionalised with a fibroblast ECM, better regulated the tendency of primary human keratinocytes to terminally differentiate when in culture. However, it was first necessary to demonstrate that fibroblasts deposited an ECM on the electrospun scaffolds. Given that SF/HNT 1% composite scaffolds supported more fibroblast growth than the other scaffolds containing HNTs, these scaffolds and SF-only scaffolds were selected for this work.

**Figure 8:**
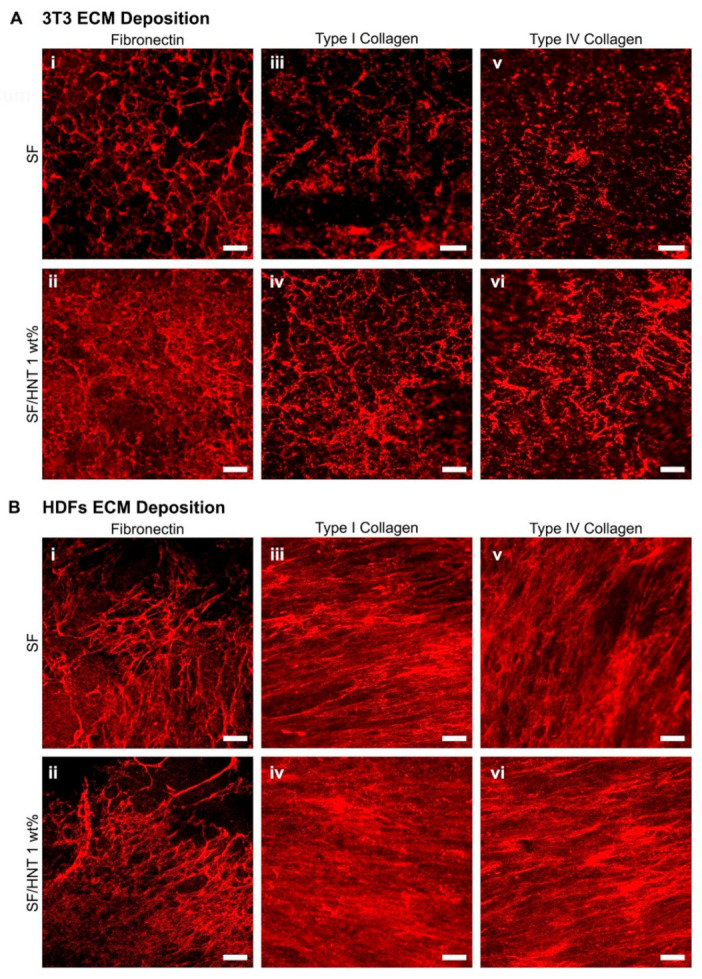
Fibroblasts deposit ECM proteins on the scaffolds. Deposition of fibronectin, collagen I, and collagen IV on SF and SF/HNT 1 wt% composite scaffolds by (**A**) 3T3 fibroblasts: cells (0.25 × 10^4^) were cultured for 7 days in DMEM (phenol red free) decellularised and the ECM fixed with 4% paraformaldehyde. (**B**) HDFs: HDFs (1 × 10^4^) were grown in DMEM (phenol red free) until day 7, decellularised and the ECM similarly fixed. Fixed scaffolds were stained with antibodies recognising fibronectin (**i**,**ii**), collagen I (**iii**,**iv**), and collagen IV (**v**,**vi**), with staining visualized using an anti-rabbit Alexa Fluor 647 antibody. Images were captured on a Nikon A1+ confocal microscope; scale bar: 50 μm.

Accordingly, 3T3 fibroblasts were seeded on SF scaffolds and SF/HNT 1 wt% composite scaffolds and cultured for 7 days, then decellularised. Immunofluorescence staining of the scaffolds revealed fibronectin, collagen I, and collagen IV were deposited by the 3T3 cells on both scaffold types (Figure 8A-i–A-vi). Still, ECM protein deposition was greater on SF/HNT 1 wt% scaffolds compared to that on the SF scaffolds. Immunofluorescence staining of primary HDFs grown on SF and SF/HNT 1 wt% composite scaffolds revealed fibronectin, collagen I, and collagen IV deposition on both scaffold types (Figure 8B-i–B-vi). The pattern and intensity of fibronectin staining were similar on the two scaffolds, and in neither case was fibronectin evenly distributed across the scaffold (Figure 8B-i,B-ii). In contrast, collagen I and collagen IV were deposited as dense, well-organised, aligned fibres (Figure 8B-iii–B-vi) that covered the scaffold surfaces. Thus, both 3T3 cells and HDFs deposit substantial ECMs on either SF or SF/HNT 1 wt% composite scaffolds, and the most pronounced differences were between the appearances of the ECMs deposited by the two cell types.

### 3.11. Primary Human Keratinocytes Remain Undifferentiated on ECM Functionalised Scaffolds

**Figure 9:**
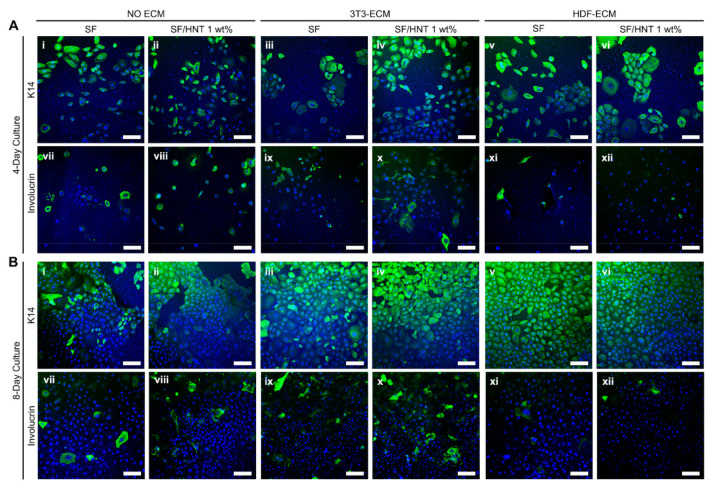
Marker expression of keratinocytes grown on SF and SF/HNT 1 wt% scaffolds. Cytokeratin 14 (K14) and involucrin expression by keratinocytes grown on the SF-based scaffolds with or without either 3T3 cell or HDF ECM for (**A**) 4 days and (**B**) 8 days. Keratinocytes (0.4 × 10^4^) were cultured in DKSFM, fixed with 4% paraformaldehyde, and stained with antibodies recognising K14 (**A-i**–**A-vi**,**B-i**–**B-vi**) and involucrin (**A-vii**–**A-xii**, **B-vii**–**B-xii**). DAPI stained nuclei (Blue); scale bar: 50 μm.

Given the above results, keratinocytes were grown for 4 days on SF and with and without a decellularised fibroblast-derived ECM coating, and the cultures were stained with an anti-K14 antibody to indicate the basal keratinocytes (Figure 9A-i–A-vi). Keratinocytes behaved differently on each scaffold. On SF and SF/HNT 1 wt% composite scaffolds, keratinocytes grew as single cells, whereas on scaffolds coated with either 3T3 cell- or HDF-derived ECM, they grew as colonies. Keratinocyte terminal differentiation was examined by staining cultures with antibodies recognising the differentiation markers, K10 and involucrin. No K10 staining was detected in cells on any of the scaffolds (data not shown), whereas the scaffolds differently triggered involucrin expression. Scaffolds coated with HDF-derived ECM had very few keratinocytes that expressed involucrin, whereas these numbers were higher on uncoated scaffolds or scaffolds coated with 3T3 cell-derived ECM (Figure 9A-vii–A-xii).

After 8 days of culture, keratinocytes on ECM-coated scaffolds formed a monolayer over the surface (Figure 9B-iii–B-vi), while on uncoated scaffolds, proliferation was slower and vacant areas were visible (Figure 9B-i,B-ii). No cells expressed K10 (data not shown), but keratinocytes expressing K14 were evident on all scaffolds (Figure 9B-i–B-vi). On SF scaffolds without a fibroblast ECM, clear and intense K14 staining was restricted to a subpopulation of cells (Figure 9B-i,B-ii), whereas K14 staining was more pronounced in keratinocytes on the 3T3 cell-derived ECM coated scaffolds, and an inclusion of 1 wt% HNTs in the scaffold resulted in a more even expression of K14 throughout the cell population (Figure 9B-iii–B-v). Scaffolds with the HDF-derived ECM had uniform K14 staining, with all keratinocytes expressing this basal cell marker regardless of the presence of HNTs. These keratinocytes were all of a similar small size, very few cells expressed involucrin, and the cell monolayers had a cobblestone-like morphology (Figure 9B-v,B-vi,B-xi,B-xii). This was particularly apparent for the keratinocytes on the HNT 1 wt% composite scaffolds functionalized with HDF ECM; keratinocytes on these surfaces formed a tight monolayer of similarly sized, small cells, indicative of an undifferentiated culture. Collectively the data suggest SF/HNT 1 wt% composite scaffolds coated with ECM from HDFs better maintained keratinocyte proliferation and an undifferentiated state.

## 4. Discussion

In this study, we found that electrospun SF/HNT composite scaffolds with 1 wt% HNTs have great potential as a biomaterial for soft tissues. Overall, they had better physical properties than SF scaffolds, being more hydrophilic. They provided a favourable microenvironment for 3T3 cell proliferation and the formation of organized muscle myotubes. Adding decellularised ECM from human dermal fibroblasts onto these composite scaffolds further expanded their applicability for tissue regeneration. In particular, the dermal fibroblast ECM coated SF/HNT 1 wt% composite scaffolds supported the proliferation of primary human keratinocytes, causing them to form an intact monolayer of small, evenly sized cells that retained a basal cell, involucrin negative, phenotype. This is an important finding, as these scaffolds are likely to be of considerable clinical benefit where the rapid expansion of primary human keratinocytes into cell sheets is required for wound healing applications.

The first part of this study involved the fabrication and characterisation of electrospun SF/HNT composite scaffolds. These scaffolds were composed of randomly oriented fibres with a smooth and generally bead-free morphology, although what appeared to be aggregates of HNTs covered by SF were apparent; these took the form of swellings or lumps in the fibres that on occasion protruded from the fibre surfaces. In addition, as the HNT content increased, there was evidence of increasing numbers of fibres with very rough, irregular surfaces. The addition of HNTs to the SF prior to electrospinning also caused the average fibre diameter to increase in accordance with the HNT loading. This is similar to the findings of others [29,46,47], where HNTs were added to polymers other than SF. The increased fibre diameter is probably due to the HNTs increasing the viscosity of the electrospun solution. Increasing solution viscosity induces increasing resistance against the jet, leading to larger fibre diameters [29,46]. EDS analyses confirmed that the HNTs were incorporated into SF fibres, while FTIR and XRD analyses indicated that β-sheet conformations were the dominant structures in methanol-treated scaffolds regardless of the HNT presence [48,49]. Previous studies reported that the transition of SF from a random coil to a β-sheet state is induced by the dehydrating effect of methanol treatment [50]. We found that HNTs had only minor effects on the overall conformation of electrospun SF fibres, and the intercalation of SF molecules into the 0.7–1 nm space between the inner and outer layers of hollow HNTs did not occur.

The incorporation of HNTs into the SF scaffolds affected their surface area. The BET surface area of SF/HNT composite scaffolds increased with increasing HNT content, possibly due to the increased surface roughness of SF/HNT composite fibres, and by the protrusion of aggregated HNTs from the fibre surfaces. Others have reported the tendency of HNTs to agglomerate as their concentration increases [51]. Our SEM images of the composite SF scaffolds revealed the number of protrusions and surface irregularities increased with increasing HNT content in accordance with the increased BET surface area values. Makaremi et al. [52] also found that incorporating HNTs increased the BET surface area of electrospun polyacrylonitrile nanofibres.

Surfaces are hydrophilic if the contact angle of a water droplet on their surface is less than 90° [53]. We found that incorporating 1 wt% HNTs improved the hydrophilicity of SF scaffolds, as the water contact angle decreased to 57.52° as opposed to 64.23° for SF-only scaffolds. The scaffolds with the highest WUC were also SF/HNT 1 wt% composite scaffolds.

The reduction in wettability of the membranes with increasing HNT loading could be due to the increase of scaffold surface roughness, as has been seen by others [53,54]. Higher surface roughness will lead to a decrease in surface energy (i.e., interfacial tension between polymer and water reduces), hence causing a higher contact angle [55]. Our SEM images of SF/HNT composite scaffolds indicated that more surface irregularities were formed as the HNT content increased, thereby yielding a rougher surface, a conclusion consistent with the BET surface area measurements. De Silva et al. [56] found that the interfacial tension between the polymer and water reduces with an increase in surface roughness, leading to materials that have more hydrophobic characteristics. Our results likely reflect a similar dominance of surface roughness over polarity, because our WUC data correlates with the hydrophilicity of the scaffolds.

The composite scaffolds containing 3 wt% HNTs exhibited a 47% and 42% increase in tensile strength and Young’s modulus, respectively, compared to the SF scaffolds. These improvements are associated with the inherent toughness of HNTs [44,46] and the efficient transfer of load from polymer matrices to the HNTs [57]. In contrast, higher HNT loadings caused a deterioration in these properties, similar to previous reports [46,57,58]. A plausible explanation is the increased agglomeration of HNTs at the higher concentrations of 5 and 7 wt%, as seen in our SEM images. These agglomerates may have hindered the interfacial adhesion of HNTs to the SF and thus reduced the effective load transfer from the SF to HNTs, resulting in detrimental mechanical properties [57,59].

The thermal stability of the scaffolds was improved with the inclusion of HNTs, but HNT agglomeration at the higher loadings yielded a slight decrease in thermal stability. This improvement in thermal stability is due to the high thermal stability of HNTs, as well as a barrier effect towards both mass and heat transport, but this only occurs when the HNTs are well dispersed [23]. Abdullah et al. [60] reported that the thermal stability of PVA/starch/HNT nanocomposites at high HNTs loadings decreased when HNTs were agglomerated, consistent with our findings.

The in vitro cytocompatibility of SF/HNT composite scaffolds was examined with several cell types. Proliferation data revealed that 3T3 fibroblast growth and viability improved on SF/HNT 1 wt% scaffolds relative to the other substrates, and fibroblasts on scaffolds containing 5–7 wt% HNTs were less spread. The increased hydrophilicity and WUC of SF/HNT 1 wt% composite scaffolds probably contributed to the better performance of these scaffolds, as increased hydrophilicity is known to assist cell attachment and proliferation [61]. Additionally, HNTs agglomerates may have negatively influenced cell adhesion and growth, as mentioned by others [57]. Overall, our results are consistent with the cytocompatibility reported for other composites containing HNTs [42,62].

The SF/HNT 1 wt% composite scaffolds best supported C2C12 cell differentiation since myotubes were longer and better aligned on these scaffolds than on any other substrate. This was not because the cells proliferated more on this surface. Substrate hydrophilicity has been proposed as a factor contributing to enhanced C2C12 myoblast differentiation [63], and Gilmore et al. [64] suggested that the surface roughness impedes the differentiation of primary myoblasts. From our results, higher hydrophilicity and the smooth surfaces of SF/HNT 1 wt% composite scaffolds appear to be beneficial for guiding the alignment and elongation of the myotubes that formed when C2C12 cells differentiated.

Given that 3T3 cell proliferation increased on SF/HNT 1 wt% composite scaffolds, it was not surprising that more collagen I and IV were deposited on these scaffolds than on SF scaffolds. Regardless of the scaffold, the deposition patterns of collagens I and IV by the HDFs, and the 3T3 fibroblasts, were very different. This contrasted with similar fibronectin deposition patterns. The 3T3 cells deposited collagens I and IV in an open mesh-like pattern, while HDFs deposited these collagens as densely aligned strands that, in places, were crossed-linked. Interestingly, others found that dressings of lyophilized SF in a β-sheet form following ethanol treatment caused increased HDF migration and promoted the expression by HDFs of the ECM proteins, fibronectin, and collagen III [65]. Collagens I and IV and fibronectin are key ECM components of skin [66]. Fibronectin has a central role in cell adhesion, migration, and differentiation, and collagen IV is a major protein of the dermal-epidermal junction and is involved in regulating keratinocyte proliferation. We, and others, have previously shown collagen IV is secreted by HDFs in culture [32,67]. We also showed the importance of ECM proteins for maintaining the proliferative phenotype of primary human keratinocytes [32], though, in that study, the HDF-derived ECM was deposited on tissue culture plastic.

Here primary human keratinocytes were cultured on decellularised ECM from either 3T3 cells or HDFs on SF or SF/HNT 1 wt% composite scaffolds, surfaces that better resemble the in vivo microenvironment than ECM on plastic. Although both scaffold types supported keratinocyte growth without an ECM, we found the addition of a decellularised fibroblast ECM markedly enhanced keratinocyte proliferation. The SF/HNT 1 wt% composite scaffolds were favoured, as on these scaffolds keratinocytes grew as colonies of small cells. This was very evident when the scaffold was coated with HDFs-derived ECM.

An exciting discovery was that when primary human keratinocytes were given a microenvironment of HDF-derived ECM on SF/HNT 1 wt% composite scaffolds, they grew without a feeder layer, and their differentiation pathways were down-regulated. As evidenced by the fact that on SF/HNT 1 wt% composite scaffolds coated with HDF-derived ECM, keratinocytes grew as tight monolayers of small, uniform cells lacking the expression of the terminal differentiation marker, involucrin. Generally, when primary human keratinocytes are grown in a serum-free medium without feeder cells, terminal differentiation limits their expansion. This can be problematic in clinical situations where rapid keratinocyte expansion for treating burns victims can be critical for minimizing scarring during wound healing and for good patient outcomes. Keratinocytes populations that are undifferentiated and of a basal cell phenotype are preferred for grafting because they are more likely to adhere to and proliferate on the wound bed. Whether keratinocytes expanded on SF/HNT 1 wt% composite scaffolds coated with HDF-derived ECM maintain their basal cell phenotype, and proliferate, following grafting onto a wound bed has not yet been examined. Still, this ongoing work will be an important indicator of the clinical applicability of this substrate.

Extracellular matrices are tissue-specific, and the ECM from one tissue best maintains cells from that same tissue. Indeed, Marinkovic et al. [68] showed the proliferation of bone marrow mesenchymal stem cells and adipose mesenchymal stem cells was best sustained when cultured on their corresponding tissue-derived ECM. Similarly, Sellaro et al. [69] found ECM from the liver better sustained the phenotype of hepatic sinusoidal endothelial cells during in vitro culture compared to an ECM derived from the urinary bladder or the small intestinal submucosa. We found that ECMs from murine 3T3 fibroblasts and HDFs differed in the quantities and the arrangements of collagen I and IV fibres, and it is likely that many minor ECM components were also qualitatively or quantitatively different. Moreover, considerably more involucrin expression occurred in keratinocytes on 3T3 fibroblast ECM than in keratinocytes on HDF-derived ECM on the same type of scaffold. Consequently, the HDF-derived ECM coated scaffolds provided a more physiologically appropriate microenvironment for maintaining primary human keratinocytes in a proliferative, undifferentiated state.

Thus, a key finding was the superior performance of the SF/HNT 1 wt% composite scaffolds. Three different cell types, namely connective tissue cells (3T3 fibroblasts), skeletal muscle myoblasts (C2C12 cells), and primary human epithelial cells (keratinocytes), performed better on this scaffold. The data further suggest that the physiochemical properties such as hydrophilicity/hydrophobicity, surface morphology, and roughness affect the cell compatibility of electrospun scaffolds comprising SF and HNTs. Moreover, functionalizing SF/HNT 1 wt% composite scaffolds by adding HDF-derived ECM promoted keratinocyte proliferation in the absence of marked cell differentiation. This finding could be exploited clinically in situations where the rapid production of keratinocytes for wound healing is critical.

## 5. Conclusions

The present study describes the successful fabrication of electrospun SF/HNT composite scaffolds. In comparison with SF scaffolds, the addition of HNTs increased thermal stability. Composite scaffolds containing 1 wt% HNTs had an increase in hydrophilitity along with an improvement in WUC. In vitro cytocompatibility of all SF/HNT composite scaffolds and particularly the SF/HNT 1 wt% composite scaffolds, was demonstrated by the viability, morphology, and proliferation of three different cell types grown on these scaffolds. In addition, the HDF-derived ECM coated SF/HNT 1 wt% composite scaffold developed in this study is an excellent substrate for the rapid in vitro expansion of undifferentiated keratinocytes. While more work is required before the scaffold can be adopted in the clinic, its potential is clear. Collectively, our data indicate that an electrospun SF/HNT 1 wt% composites scaffold is a promising material for soft tissue engineering applications.

## Figures and Tables

**Table 1:** BET Surface Area Data of—SF scaffolds and SF/HNT composite scaffolds.

Sample	BET Surface Area (m^2^/g)
SF	4.47 ^1^
SF/HNT 1 wt%	5.14
SF/HNT 3 wt%	8.06
SF/HNT 5 wt%	8.43
SF/HNT 7 wt%	9.06

^1^ Representative data from two replicate experiments.

**Table 2:** Static contact angles of SF scaffolds and SF/HNT composite scaffolds.

Sample	Water Contact Angle (Degrees)
SF	64.23°± 4.01°
SF/HNT 1 wt%	57.52° ± 6.73° *
SF/HNT 3 wt%	65.73° ± 7.29°
SF/HNT 5 wt%	66.33° ± 5.46°
SF/HNT 7 wt%	70.76° ± 4.82° *

Mean and SD are shown. Statistical analyses: ANOVA followed by Tukey’s test. * = *p* ≤ 0.05.

**Table 3:** Thermal decomposition/stability of SF scaffolds, HNT particles, and SF/HNT composite scaffolds.

Sample	TGA		DTG		
	T _10%_^a^	T _50%_^a^	T_d1_^c^	T_d2_^c^	T_d3_^c^
SF	201.65 ^b^	395.3	68.22	222.41	290.65
HNT particles	–	–	–	–	499.56
SF/HNT 1 wt%	230.84	407.6	70.97	231.37	311.54
SF/HNT 3 wt%	240.63	412.68	70.85	234.35	312.42
SF/HNT 5 wt%	239.88	409.3	69.9	228.26	309.26
SF/HNT 7 wt%	234.52	408.4	68	226.49	308.57

^a^ The decomposition temperatures at weight losses of 10 and 50% were labelled as T10% and T50%, respectively, and were determined from TGA curves (Figure 5A). ^b^ Temperature unit is degrees Celsius. ^c^ Data are temperature peaks from Figure 5B.

## Data Availability

Data are on the Curtin University server and in laboratory notebooks stored in the School of Civil and Mechanical Engineering, Curtin University.

## Supplementary Materials

The following supporting information can be downloaded at: https://www.mdpi.com/article/10.3390/polym14153004/s1, Figure S1: Fibre abnormalities in scaffolds with high HNT content.

## Abbreviations

Bet, Brunauer−Emmet−Teller; DAPI, 4′,6-diamidino-2-phenylindole; DKSFM, defined keratinocyte serum-free medium; DTG, derivative thermogravimetric; ECM, extracellular matrix; EDS, energy-dispersive X-ray spectrometer; FE-SEM, field emission scanning electron microscope; FTIR, Fourier transform infrared spectroscopy; HDFs, human dermal fibroblasts; HNTs, halloysite nanotubes; SD, standard deviation; SEM, Scanning electron microscope; SF, silk fibroin; TGA, thermogravimetric analysis; WUC, water uptake capacity; XRD, X-ray diffraction.
