# Supplementary material for: Fabrication and Evaluation of Electrospun Silk Fibroin/Halloysite Nanotube Biomaterials for Soft Tissue Regeneration
Source: Polymers (Basel). 2022 Jul 25;14(15):3004. doi: 10.3390/polym14153004 (PMC9332275; doi:10.3390/polym14153004)
Supplement: Supplementary file 1 [file polymers-14-03004-s001.zip › polymers-1752477-supplementary.pdf]

Supplementary Information:

Fabrication and Evaluation of Electrospun Silk Fibroin/Halloysite Nanotube Biomaterials for Soft Tissue Regeneration

Soheila Mohammadzadehmoghadam[1,2], Catherine F. LeGrand[2,3], Chee-Wai Wong[2,3], Beverley F. Kinnear[2,3], Yu Dong[1,*], and Deirdre R. Coombe[2,3,*]

[1] School of Civil and Mechanical Engineering, Curtin University, Bentley WA 6102 Australia; sm.mohamadzade@gmail.com
[2] Curtin Health Innovation Research Institute, Faculty of Health Sciences, Curtin University, Bentley, WA 6102 Australia
[3] Curtin Medical School, Pharmacy and Biomedical Sciences, Curtin University, Bentley, WA 6102 Australia
* Correspondence: Y.D. Y.Dong@curtin.edu.au; D.R.C. D.Coombe@curtin.edu.au

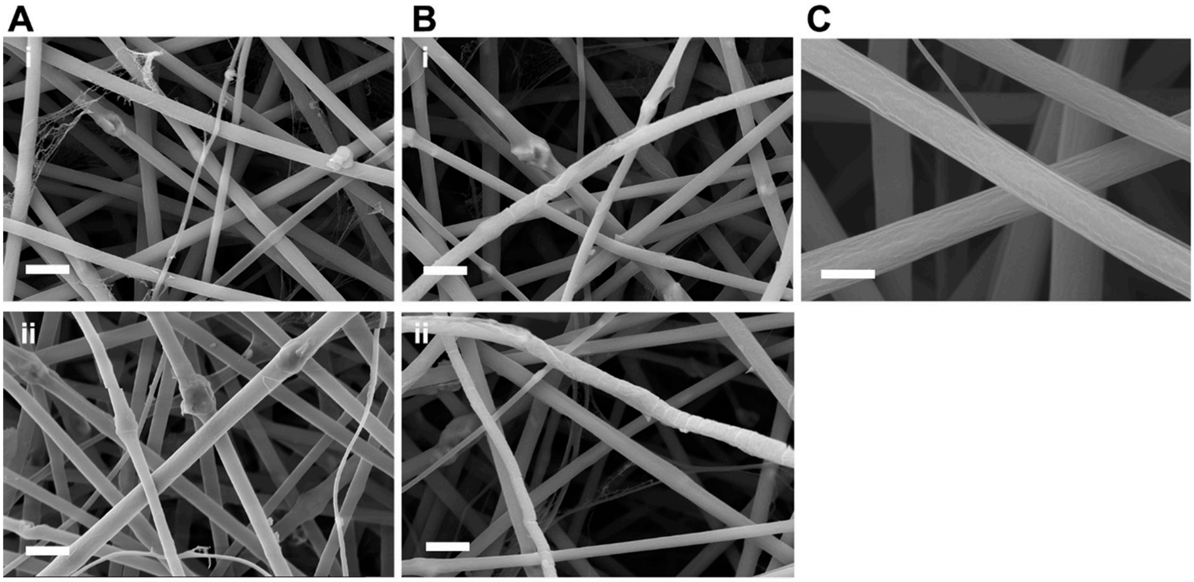

Figure S1. Fibre abnormalities in scaffolds with high HNT content (A) SF/HNT 5% wt% and (B) SF/HNT 7% wt% scaffolds; (i & ii) are images from different replicate scaffolds; Scale bar: 1 µm. (C) SF scaffold, no HNT; Scale bar 400 nm
